# Genome Sequence of *Paenibacillus polymyxa* ATCC 12321, a Promising Strain for Optically Active (*R*,*R*)-2,3-Butanediol Production

**DOI:** 10.1128/genomeA.00572-13

**Published:** 2013-08-01

**Authors:** Ying-Jia Tong, Xiao-Jun Ji, Lu-Gang Liu, Meng-Qiu Shen, He Huang

**Affiliations:** State Key Laboratory of Materials-Oriented Chemical Engineering, College of Biotechnology and Pharmaceutical Engineering, Nanjing University of Technology, Nanjing, People’s Republic of China

## Abstract

*Paenibacillus polymyxa* is a potential strain for (*R*,*R*)-2,3-butanediol production. Here, we report an annotated draft genome sequence of *P. polymyxa* strain ATCC 12321, which contains 4,429 protein-coding genes and 49 structural RNAs. This genome sequence provides a genetic basis for a better understanding of the mechanism for the accumulation of highly optically active (*R*,*R*)-2,3-butanediol.

## GENOME ANNOUNCEMENT

2,3-Butanediol (2,3-BD) is one of the promising bio-based bulk chemicals due to its extensive industrial applications (1–4). Furthermore, due to its two chiral centers, the molecule of 2,3-BD contains three stereoisomers, namely (*S*,*S*)-, (*R*,*R*)-, and *meso*-2,3-BD (5). Among the three stereoisomers, the optically active (*R*,*R*)-2,3-BD has special applications in the asymmetric synthesis of valuable chiral specialty chemicals (6). Various bacteria were investigated to produce 2,3-BD (1, 2), while only *Paenibacillus polymyxa* (formerly *Bacillus polymyxa*) had the ability to produce (*R*,*R*)-2,3-BD at over 98% optical purity (7–9). Generally, in addition to (*R*,*R*)-2,3-BD, the strain produced a small amount of *meso* stereoisomer at the same time (1, 5), and the ratio of these two stereoisomers was usually affected by the environmental conditions, such as aeration and pH (10). However, the mechanism is not clear. Furthermore, the reported mechanisms for the two-stereoisomer formation in *P. polymyxa* were still controversial (5). Studies being performed to elucidate these detailed mechanisms are now focused on the analysis and annotation of a complete genome sequence. To date, three complete genome sequences and two draft genome sequences of *P. polymyxa* strains have been stored in the NCBI database, but these *P. polymyxa* strains all belong to plant-growth-promoting rhizobacteria, not the (*R*,*R*)-2,3-BD-producing strain. Therefore, sequencing *P. polymyxa* ATCC 12321, which is a promising strain for optically active (*R*,*R*)-2,3-BD production (8), will not only enrich the genome sequence database of *P. polymyxa*, but it also will help to elucidate the genetic background of this useful strain.

Here, we present the draft genome sequence of strain ATCC 12321, obtained using the Illumina HiSeq 2000 next-generation DNA platform. Sequencing was performed by Shanghai Majorbio Pharm Technology Co., Ltd., with a paired-end library. The reads were trimmed and *de novo* assembled with SOAP*denovo* (http://soap.genomics.org.cn/) v1.05. Open reading frames (ORFs) were identified by the program Glimmer 3.0 (http://ccb.jhu.edu/software/glimmer/index.shtml). These ORFs were further annotated by comparison with the NCBI nr database and BLASTp (blast 2.2.24). The rRNAs were predicted by RNAmmer (11), and tRNAs were predicted by tRNAscan (12).

The draft genome sequence of strain ATCC 12321 comprises 4,136,795 bp, which is assembled into 92 contigs. The *N*_50_ quality measurement of the contigs is 172,434 bp and the largest contig assembled is approximately 587,692 bp. It has a G+C content of 46.004%. There are 4,429 predicted protein-coding sequences in the genome sequence. The chromosome has 1 rRNA operon and 48 tRNAs, as predicted by RNAmmer and tRNAscan, respectively.

The genome sequence of *P. polymyxa* ATCC 12321 serves as a basis for further investigation of the molecular basis of its high optically active (*R*,*R*)-2,3-BD production ability. Relatively detailed annotations will further reveal the mechanism for the (*R*,*R*)- and *meso*-stereoisomer accumulation ratio vitiations in response to different environmental conditions.

### Nucleotide sequence accession numbers. 

This whole-genome shotgun project has been deposited at DDBJ/EMBL/GenBank under the accession no. ARYD00000000. The version described in this paper is the first version, accession no. ARYD01000000.
